# Airbrushed Polysulfone (PSF)/Hydroxyapatite (HA) Nanocomposites: Effect of the Presence of Nanoparticles on Mechanical Behavior

**DOI:** 10.3390/polym14040753

**Published:** 2022-02-15

**Authors:** Monireh Moradienayat, Dania Olmos, Javier González-Benito

**Affiliations:** Departamento de Ciencia e Ingeniería de Materiales e Ingeniería Química, Universidad Carlos III de Madrid, IQMAAB, Avda. de la Universidad 30, 28911 Leganés, Spain; mmoradie@ing.uc3m.es

**Keywords:** polysulfone, hydroxyapatite, nanocomposites, interphases, mechanical properties

## Abstract

Nanocomposite films of polysulfone (PSF)—hydroxyapatite (HA) were prepared with a commercial airbrush. Structural, thermal, and mechanical characterization allows obtaining new information to understand the role of the nanofiller–polymer matrix interphase in the final performance of these materials in relation to its possible applications in the restoration of bones. Fourier-transform infrared spectroscopy shows that there are hardly any structural changes in the polymer when adding HA particles. From thermal analysis (differential scanning calorimetry and thermogravimetry), it can be highlighted that the presence of HA does not significantly affect the glass transition temperature of the PSF but decelerates its thermal degradation. All this information points out that any change in the PSF performance because of the addition of HA particles cannot be due to specific interactions between the filler and the polymer. Results obtained from uniaxial tensile tests indicate that the addition of small amounts of HA particles (1% wt) leads to elastic moduli higher than the upper bound predicted by the rule of mixtures suggesting there must be a high contribution of the interphase. A simple model of the nanocomposite is proposed for which three contributions must be considered, particles, interphase and matrix, in such a way that interphases arising from different particles can interact by combining with each other thus leading to a decrease in its global contribution when the amount of particles is high enough. The mechanical behavior can be explained considering a balance between the contribution of the interphase and the number of particles. Finally, a particular mechanism is proposed to explain why in certain nanocomposites relatively high concentrations of nanoparticles may substantially increase the strain to failure.

## 1. Introduction

When they are in service, certain natural materials need to be repaired or even substituted as a consequence of failure or degradation phenomena. However, in many cases, the use of original natural material is not profitable because of its scarcity in nature or difficulties in its extraction or processing. Therefore, developing new synthetic materials that can mimic most of the properties of the natural material is crucial [1]. As an example of natural materials, bones are receiving much attention because of their implication in tissue engineering and regenerative medicine [2].

Bones have distinctive properties, which result from their unique chemical composition and precise structural design. Bone is one of the tissues with more interest from the point of view of research in materials science and engineering because of two main reasons [3]. First, because the bone is the most transplanted tissue (after blood) [4], and therefore, great efforts should be focused within the frame of tissue engineering (TE) and on the design and preparation of new materials that can be used to restore damaged tissue [5]. Secondly, the investigation of materials that can mimic bone composition, structure, and mechanical properties is essential from the point of view of other disciplines, such as conservation and restoration of historical bones [6,7], in this sense, another strategy considers the modification of hydroxyapatite with the aid of radiation [8,9]. It is noteworthy to mention the research carried out by using X-ray radiation to modify the properties of hydroxyapatite and its interaction with the organic matrix in a composite material for bone tissue engineering applications [9]. In this work, different doses of X-ray were used to modify cortical bone. The changes at the molecular scale, studied by FTIR spectroscopy, were correlated with variations in mechanical properties of the bone samples [9].

Among the different bioceramics, hydroxyapatite (HA) and calcium phosphates have been proposed as ideal components of materials for bone tissue applications [6,10,11,12]. The use of hydroxyapatite in vivo applications is well known due to its close structure and chemical composition to that of natural bone [13,14,15]. Besides, hydroxyapatite is used in medical applications such as the treatment of bone defects [13] and tissue regeneration due to its biocompatible properties [16,17]. However, when other applications such as restoration and consolidation of historical bones are considered, the use of just the bioceramic particles may not be enough. For example, important difficulties can be found when treating the surfaces to be restored and poor dimensional integrity is expected because of the lack of effective binding between particles. A possible approach to overcome this may be the use of a ligand material that, in addition, helps to conveniently spread out the restoring material, effectively coating or filling the bone substrate. Among the existing materials, several thermoplastics can have adequate properties to be used as ligand or matrices in polymer/hydroxyapatite composites for restoring bones [18,19]. In fact, some of them have thermomechanical properties not very different from those of bones. Besides, they can be melted or dissolved to subsequently be easily spread out over any surface.

One example of thermoplastic polymer with convenient characteristics for the above-mentioned applications is polysulfone, PSF. It has good thermal and chemical stability and high mechanical strength [20,21]. Besides, PSF is biocompatible and has been used for bio-related applications such as bone prosthetic material in orthopedics and implants, with promising results in animal models [22] or in dentistry applications [23]. However, in certain cases, polysulfone by itself cannot accomplish some mechanical, aesthetic, and optical properties that allow it to be an adequate substitute for bone-like tissues; therefore, its modification by the addition of fillers may be a good solution [24].

On the other hand, apart from the properties of hydroxyapatite already mentioned, its use as a filler in the form of nanoparticles might also be interesting. It is well known that the nanoscale character may contribute to the appearance of improved properties or even new ones, for instance, enhanced mechanical properties and good optical properties if nanoparticles are properly dispersed.

In the particular case of historical bones, up to now, different methods of application of consolidates have been used for the restoration, brushing, immersion in solutions, or impregnation with consolidating agents, for instance [23,25,26,27]. However, historical bones might present low mechanical consistency and it would be recommended to use less aggressive consolidation treatment methods. In the present work, the use of a simple commercial airbrush to softly treat any surface with a polymer-based nanocomposite is proposed. Airbrushing is a versatile method that can be easily used to coat and treat degraded surfaces in situ by spraying the polymer solution or suspension on the desired surface, controlling the amount and, consequently, the thickness of the material deposited. Besides, the use of airbrushing in particular and solution spraying methodologies in general have been revealed as good methods to produce polymer-based nanocomposites with a uniform dispersion of nanoparticles [28,29,30,31,32], which is necessary if homogeneous materials in terms of their properties are required.

In this research, polymer nanocomposites in the form of films based on PSF filled with HA nanoparticles are prepared using solution spraying by airbrushing as a potential method for restoring bones. The materials prepared are characterized in terms of structure, morphology, and thermal and mechanical properties to study the effect of HA nanoparticles addition on the final material performance. Finally, in order to give an understandable explanation about the nanoparticles’ influence, a simple model of the nanocomposite is given and a theoretical mechanism of the mechanical behavior is proposed.

## 2. Materials and Methods

### 2.1. Materials

Polysulfone (PSF) supplied by Sigma-Aldrich (average number and weight molar masses, M_n_ ~ 16,000 by MO and M_w_ ~ 35,000 g·mol^−1^, respectively, density 1.24 g·mL^−1^ at 25 °C) was used as a polymer matrix. On the other hand, as a nanofiller, hydroxyapatite nanoparticles, HA, with a diameter of ≤200 nm were used (reference number 1002598785, Sigma-Aldrich). Finally, to prepare the polymer solutions and suspensions to be blow spun, tetrahydrofuran, THF (purity 99.9%, Sigma-Aldrich, St. Louis, MO, USA) was used as the solvent.

**Figure 1:**
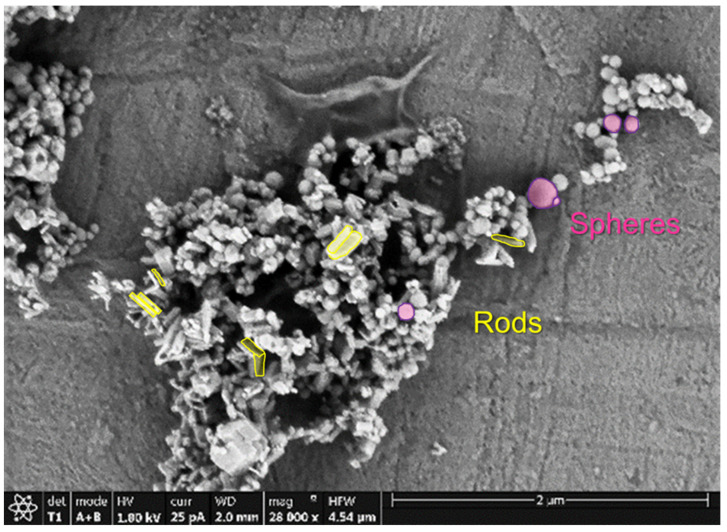
SEM micrographs of the commercial particles of hydroxyapatite (HA) observed at 28,000×.

The structure of the commercial nanoparticles was checked by X-ray diffraction (Figure S1). The diffraction pattern obtained for the HA nanopowder was in good agreement with that given by the Joint Committee on Powder Diffraction Standard, JCPDS (code: 9-432). Besides, scanning electron microscopy (SEM) micrographs of hydroxyapatite nanoparticles were obtained to ensure the specified size given by the supplier (Figure 1). As can be observed, spherical and rod-like prismatic particles with sizes in the range of 20–220 nm could be found. In Figure S2, particle size distributions obtained for the commercial hydroxyapatite nanoparticles are presented (diameters of the spherical particles can be seen in Figure S2a while lengths of long and short axes in the rod-like particles are shown in Figure S2b,c, respectively). The average size of the nanoparticles matches well the information provided by the supplier (<200 nm).

### 2.2. Sample Preparation

Nanocomposites made of PSF filled with HA, PSF/HA, in the form of films were prepared with different concentrations of particles (0%, 1%, 2%, 5%, and 10% wt). To obtain the final films by airbrushing, it was first necessary to prepare the corresponding polymer solution, which was either a sample of neat polymer (0% wt of HA) or, in the case of composites, suspensions of HA nanoparticles in the polymer solution. The solutions were obtained dissolving 0.5 g of PSF in 10 mL of THF (5% g/mL). The nanocomposites were prepared from a suspension made by mixing a solution of 0.5 g of PSF in 7 mL of THF with 3 mL of THF (to keep constant the concentration of the PSF solution) containing the required suspended amount of HA nanoparticles as to finally have a nanocomposite with a particular concentration of nanofiller. To facilitate disaggregation of HA nanoparticles, the 3 mL suspension of HA was sonicated (30 min at room temperature) before being added to the PSF solution. After stirring the HA suspensions in the PSF solution for 15 min, they were poured into the 5 cm^3^ reservoir of a commercial airbrush to subsequently spray them on the surface of a flat aluminum foil. The main airbrush conditions were: (a) air as an ejecting gas at 2 bars of pressure; (b) room temperature (22 °C); and (c) working distance (distance from the nozzle to the collector) of 5 cm. All films prepared were stored in a desiccator.

### 2.3. Characterization

Structural and morphological characterization of the commercial hydroxyapatite nanopowder was done by X-ray diffraction (XRD) and field emission scanning electron microscopy (FESEM), respectively. X-ray diffraction experiments were performed in a Phillips X’Pert diffractometer in the range 2θ = 20–70° using the wavelength of K_α1_ (Cu) (λ = 0.15406 nm). The working conditions were set at 40 kV and 40 mA. To study the morphology of the nanoparticles and their distribution in the polymer nanocomposites, a TENEO field emission scanning electron microscope, FESEM (FEI), was used. In the case of inspecting the morphology of the particles, the microscope was used in the FSEM mode applying an acceleration voltage of 1.00 kV and taking the signal coming from secondary and backscattered electrons using an ETD and Trinity T1 (working in the A + B Z-contrast mode) detectors, respectively. In order to avoid electrostatic charge accumulation, the HA particles were carbon-coated by evaporation using a Leica EM ACE200 low vacuum coater. On the other hand, the nanocomposites were inspected by the microscope in the STEM mode (scanning transmission electron microscopy), applying a voltage of 22.00 kV and using a high angular annular dark field (HAADF) detector. For the visualization of the nanocomposites by STEM, the samples were placed on copper grids (Formvar Carbon Film on 400 square mesh, FCF400-Cu). Microanalysis by energy-dispersive X-ray spectroscopy, EDS, was also carried out in order to confirm the location of the different phases and their local concentration.

To study the influence of the presence of HA nanoparticles in the polymer structure of the PSF polymer, the PSF/HA nanocomposites were characterized by Fourier-transform infrared (FTIR) spectroscopy using attenuated total reflectance, ATR. The spectra were recorded at room temperature in a Shimadzu Affinity 1 spectrometer equipped with a Golden Gate ATR accessory (diamond window), from 600 to 4000 cm^−1^ with a resolution of 4 cm^−1^ and averaging 32 scans. The software OMNIC ESP v5.1 (Nicolet) was used for the numerical treatment of the spectra.

The thermal behavior of the materials was studied using differential scanning calorimetry, DSC, and thermogravimetric analysis, TGA. DSC experiments were carried out in a Mettler Toledo DSC822^e^ instrument (Madrid, Spain) under nitrogen atmosphere using the following thermal cycle: (i) a heating scan from 60 °C to 220 °C at 10 °C·min^−1^; (ii) an isothermal step at 220 °C for 10 min; (iii) a cooling scan from 220 °C to 60 °C at 10 °C·min^−1^; and (iv) a second heating scan from 60 °C to 220 °C at 10 °C·min^−1^. The two heating scans will allow studying the effect of the processing method plus the presence of nanoparticles (first heating scan) and, just the presence of nanoparticles (second heating scan) after erasing the processing history. Thermal degradation of the samples was studied by TGA. The experiments were carried out in a TGA-SDTA 851 Mettler Toledo thermobalance (Madrid, Spain) by heating from 30 °C to 750 °C at a heating rate of 10 °C·min^−1^ under a nitrogen atmosphere with a gas flow of 20 mL·min^−1^. The TGA curves were then processed to also obtain the differential thermogravimetric analysis (DTGA) curves.

**Figure 2:**
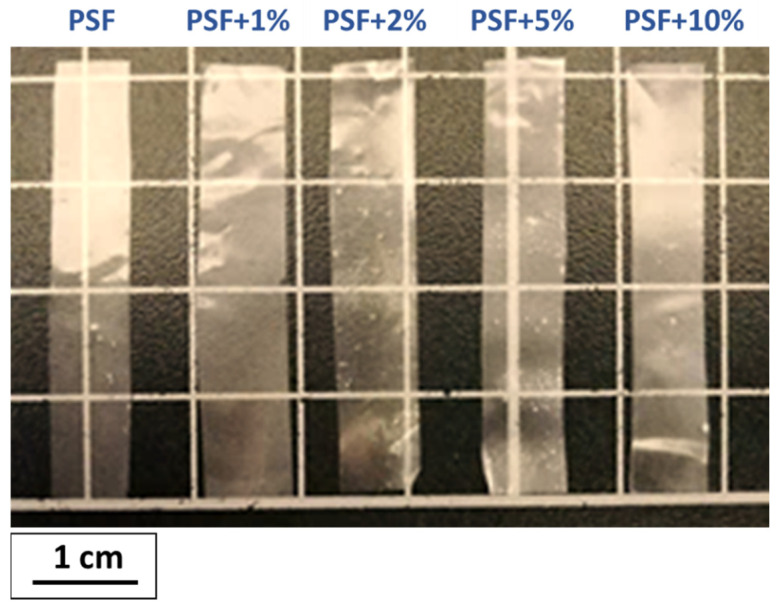
Photograph of the specimens prepared for mechanical tests.

Mechanical characterization of the films was performed using a testing machine Microtest DT/005/FR (Microtest S.A., Madrid, Spain) with a load cell of 50 N. Six specimens of each sample were tested in a uniaxial tensile configuration using a loading rate of 1 mm·min^−1^. The dimensions of the specimens were 4 cm length, 6 mm width, and an average thickness of 40 µm (Figure 2). The average values of the mechanical parameters such as elastic modulus, tensile strength, total deformation at failure, and energy absorbed were extracted from the analysis of the tensile tests curves.

## 3. Results

### 3.1. Morphology

**Figure 3:**
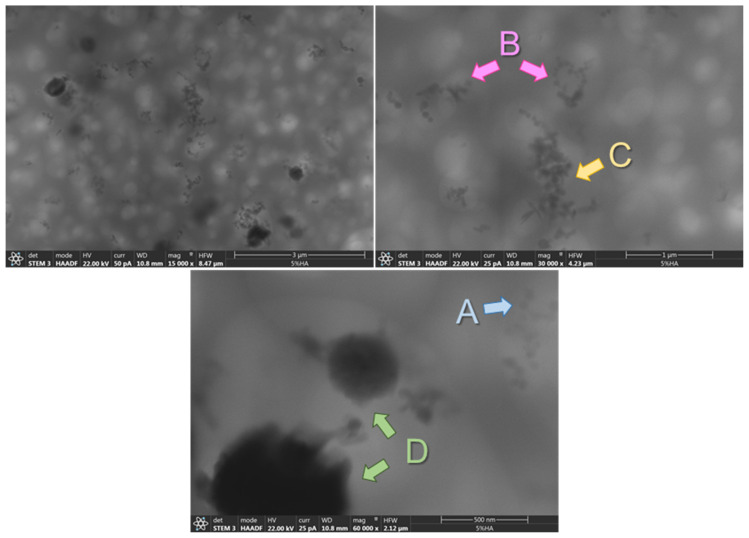
STEM images at different magnifications of the PSF-based composite with 5% wt of HA nanoparticles (15,000×; 30,000× and 60,000×).

As can be observed in Figure 2, the materials prepared show high transparency regardless of the concentration of HA particles added. This result suggests a quite uniform dispersion of the nanoparticles at least from a macroscopical point of view. In Figure 3, as an example, STEM images of the PSF-based composite with 5% wt of HA nanoparticles are shown. In any case, regardless of the concentration of hydroxyapatite, a continuous clearer region (less contrast) is observed that can be associated with regions with lower density and therefore corresponds to the PSF neat polymer and dark regions that point out the presence of HA nanoparticles. On the other hand, among the darker regions, four different features can be distinguished: (a) isolated particles (pointed out on the images with the letter A); (b) small aggregates (pointed out on the images with the letter B); (c) large aggregates (pointed out on the images with the letter C); and (d) spherical-like agglomerates (pointed out on the images with the letter D).

**Figure 4:**
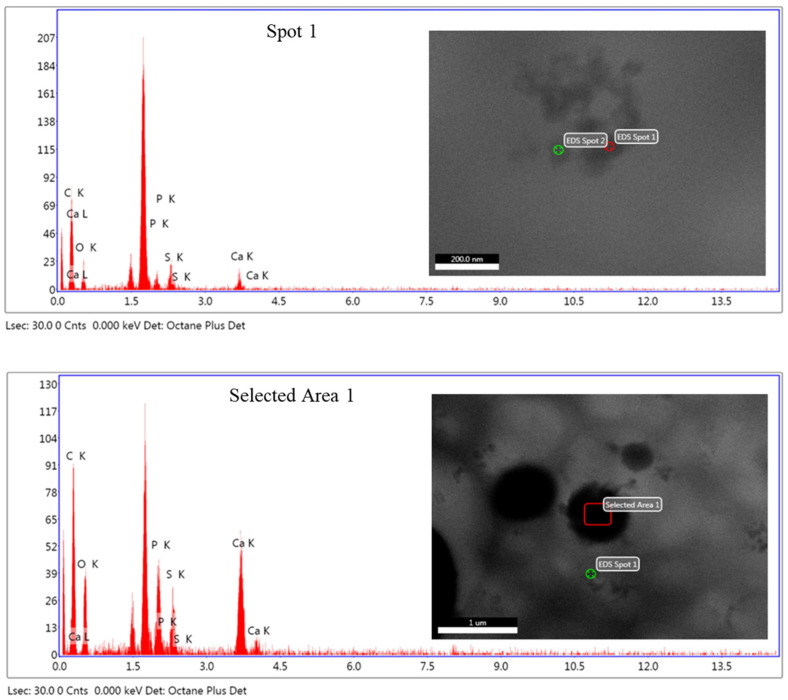
X-ray spectra were taken from a large aggregate (**top**) and an agglomerate (**bottom**).

In every case, elemental microanalysis was carried out to obtain local information about the relative amount of HA used for the purpose of the calcium to sulfur signal ratio (Ca/S). In Figure 4, two X-ray spectra taken from one aggregate and one agglomerate, respectively, are shown. In both cases, the appearance of HA is clear if the signal coming from Ca and P are considered. Furthermore, as expected, in the case of the X-ray spectrum taken at the agglomerate (Figure 4, bottom) a higher ratio of Ca/S is observed evidencing a higher accumulation or density of HA nanoparticles.

Although the dimensions between aggregates and agglomerates might not be different, they differ from the density of particles clearly evidenced by the darkness observed in the images and the relative amount of Ca. In Figure 3, details of an isolated nanoparticle, small and large aggregates, are shown.

**Figure 5:**
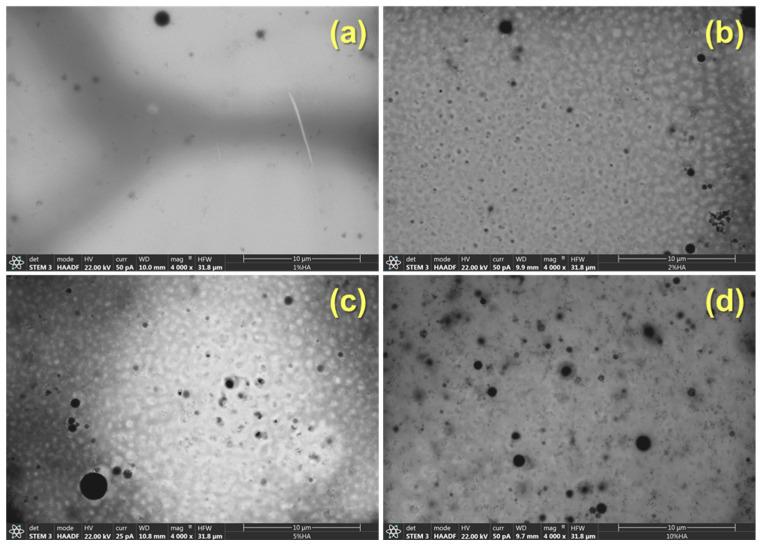
STEM images at low magnification of the PSF/HA nanocomposites under study: (**a**) 1%; (**b**) 2%; (**c**) 5%; and (**d**) 10% wt of HA, respectively.

**Figure 6:**
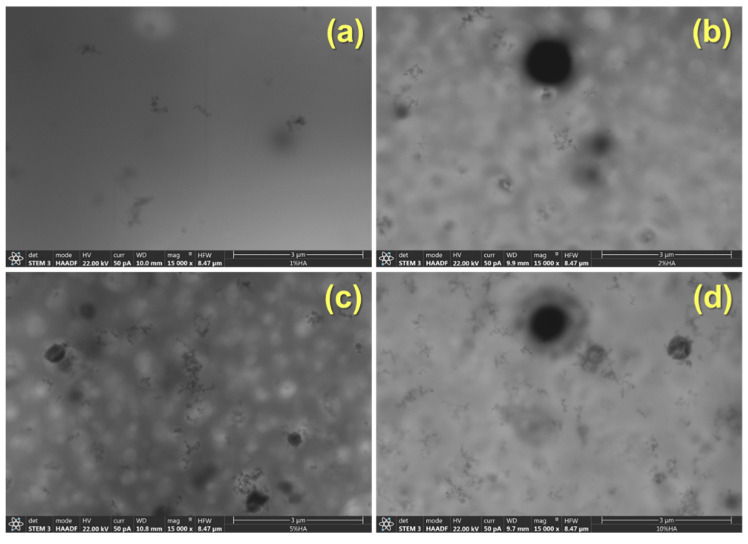
STEM images at medium magnification of the PSF/HA nanocomposites under study: (**a**) 1%; (**b**) 2%; (**c**) 5%; and (**d**) 10% wt of HA, respectively.

Comparing the materials as a function of the concentration of HA, it can be clearly seen that there is a change in the contribution of the different morphological features to the general morphology (Figure 5 and Figure 6). With 1% of HA, there are mainly isolated particles and small aggregates (Figure 5 and Figure 6). When 2% of HA nanoparticles are added, although some agglomerates can be seen, the main contribution is due to large aggregates and finally, with 5% and 10%, the morphology is mainly constituted by agglomerates and large aggregates (Figure 5 and Figure 6).

### 3.2. Structural Characterization

**Figure 7:**
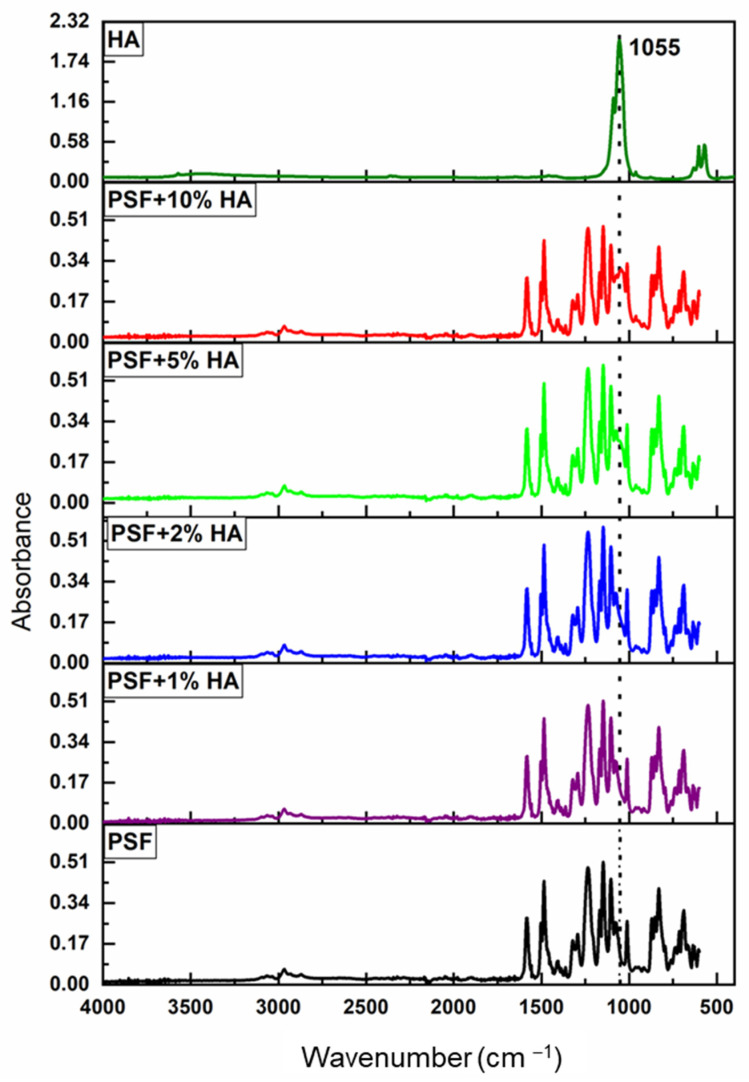
ATR-FTIR spectra for PSF/HA films at different % wt of HA. FTIR spectra of HA and PF are included for reference purposes.

In Figure 7, ATR-FTIR spectra (mid infrared) of HA nanoparticles and PSF-based materials with different HA particle contents are shown. In Table 1, the main absorption peaks and the corresponding band assignments were collected. At the top of Figure 7, the characteristic absorption bands of HA corresponding to (PO_4_)^3−^ and OH^−^ can be seen at 1055 cm^−1^ and 3500 cm^−1^, respectively [33]. On the other hand, at the bottom of Figure 7, the main characteristic bands of the PSF can be identified, for instance, at 1020 cm^−1^ and 1103 cm^−1^ the peaks associated to the aromatic C–H in-plane bending vibrations, at 1151 cm^−1^ the stretching vibrations of the group O=S=O, at 1244 cm^−1^ the asymmetric stretching vibration of the C−O–C, and at 1292 cm^−1^ and at 1322 cm^−1^ the bands attributed to the S=O symmetric and asymmetric stretching vibrations. Lastly, the peaks found in the region 1490 cm^−1^–1585 cm^−1^ correspond to the aromatic stretching vibrations [34,35,36,37]. As can be seen, there is only one clear variation in the PSF spectrum with the addition of HA, the absorbance of the band centered at 1055 cm^−1^ increases proportionally as the concentration of nanofiller increases.

A more careful analysis of the FTIR spectra of Figure 7 is given in Figure S3a where there are no important changes in terms of band shifts or absorbance ratios. These small variations observed indicate the lack of strong nanoparticle–polymer interactions. However, as expected, there is only a more significant change of about 30% for the absorbance ratio between the PSF bands close to the intense band of HA at 1055 cm^−1^, A_1079_/A_1073_ (Figure S3b). In particular, there is a general trend with a decrease in the absorbance ratio A_1079_/A_1073_ simply because the peak at 1073 cm^−1^ is more affected by the near presence of the HA band. In spite of the above, it is noteworthy that absorbance ratio A_1079_/A_1073_ increased slightly for 1% HA and decreased for higher loadings. This result points out that for small concentrations of HA (less than 2%), more favorable interactions between nanoparticles and polymer might occur probably because of more uniform dispersion of nanoparticles in the polymer matrix. If uniform dispersion of isolated nanoparticles is obtained, a larger available surface of HA is expected and, consequently, interactions between nanoparticles and polymer are favored. On the other hand, at a certain nanoparticle concentration, the interphases generated around the particles can overlap each other and the effectiveness in the polymer–particle interactions would be lower. Furthermore, when the concentration of particles is high enough, above 1%, particle aggregates and agglomerates can be formed, thus highly reducing the available surface of HA and consequently the effectiveness of the polymer–particle interactions.

### 3.3. Thermal Characterization

**Figure 8:**
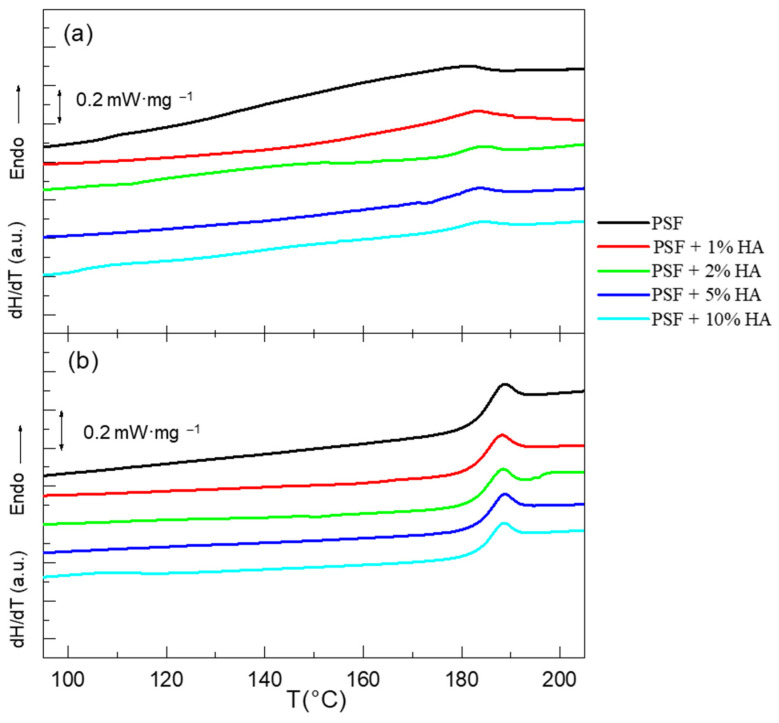
DSC traces corresponding to the (**a**) first and (**b**) second heating scan of PSF, PSF + 1%HA, PSF + 2%HA, PSF + 5%HA, and PSF + 10%HA.

In Figure 8, the DSC thermograms corresponding to the first and second heating scans of the materials under study are represented. In the first scan (Figure 8a), the thermal behavior of the “as processed” samples is considered; therefore, the thermal response of PSF should reflect the effect of the airbrushing, the effect of the presence of the HA nanoparticles, and a combination of both. As can be seen in Figure 8, the DSC traces of PSF-based materials only present a remarkable change in the heat capacity at approximately 180 °C (Table 2), which corresponds to the glass transition of polysulfone, in good agreement with other published results [38]. During the first heating scan (Figure 8a), the thermal transition occurs smoothly in a wide temperature interval, showing a very small enthalpic relaxation. This result indicates that PSF chains are arranged in an extremely poor order with high heterogeneity, probably caused by the airbrushing process. Besides, it is possible to see that the glass transitions of the materials occur over a narrower temperature interval the higher the nanoparticles concentration is. This is noticeable when comparing the curve of pure polysulfone with those of PSF + 5% HA or PSF + 10% HA. In general, shorter temperature ranges for a certain thermal relaxation indicate higher homogeneity at a local scale for the dynamics of the polymer. Therefore, this result would point out that the presence of particles enhances the polymer chains order, as it was recently described for a polymer nanocomposite formed by PMMA filled with TiO_2_ nanoparticles [39] heating scan; the polymer reaches temperatures well above the viscous flow and full polymer chain relaxation occurs. After that, when the materials are cooled down at a slow rate, enough of a higher polymer chain order can be attained which must be the consequence of the appearance of the enthalpic relaxation observed during the second heating scan. The transition temperature, T_g_, measured at the inflection points, is approximately 186 °C for all the samples (Table 2), meaning that the presence of the HA nanoparticles does not exert any remarkable effect on the polymer relaxation at the glass transition of PSF, in line with the tiny changes observed in the FTIR-ATR spectra.

Figure 8b displays the DSC thermograms of the samples for the second heating scan. After erasing the thermal and processing history of the samples, a well-defined change in the heat capacity of the samples is observed for the glass transition, showing, in addition, the typical peak associated with the so-called enthalpic relaxation at the end of the first.

As can be seen, the T_g_ of the materials for the second heating scan is higher, ~186 °C, than for the first one, ~180 °C (Figure 8 and Table 2). These results clearly suggest that the main factor affecting the dynamics of the polymer is the processing (airbrushing) more than the presence of HA nanoparticles. Therefore, the cooling process must exert an annealing effect, inducing higher polymer chain order and consequently favoring intermolecular interactions, which in turn, would lead to higher temperatures to disrupt the polymer structure.

Another remarkable difference between the first and second heating scan is the change in C_p_, ΔC_p_. In the first case, ΔC_p_ for the neat PSF is more than twice that of any of the PSF/HA composites; however, in the second heating scan, ΔC_p_ is almost constant regardless of the concentration of nanoparticles (Table 2). Therefore, it can be concluded that the presence of nanoparticles conditions the homogeneity of the PSF prepared by airbrushing, while after erasing the thermal and processing histories, the presence of nanoparticles does not seem to play any role in the thermal relaxation of PSF at the T_g_, having the polymer chains more homogeneous to local environments regardless of the concentration of HA nanoparticles.

The enthalpic relaxation of the materials was also extracted from the DSC thermograms obtained during the second heating scan, ΔH_relax_ (Table 2). In the first heating scan, almost no enthalpic relaxations are observed. When cooling the materials from the melt at a moderate rate (10 °C·min^−1^), a certain order in the PSF chains arrangement can be obtained which may cause the enthalpic relaxation phenomena observed in the second heating scan.

**Figure 9:**
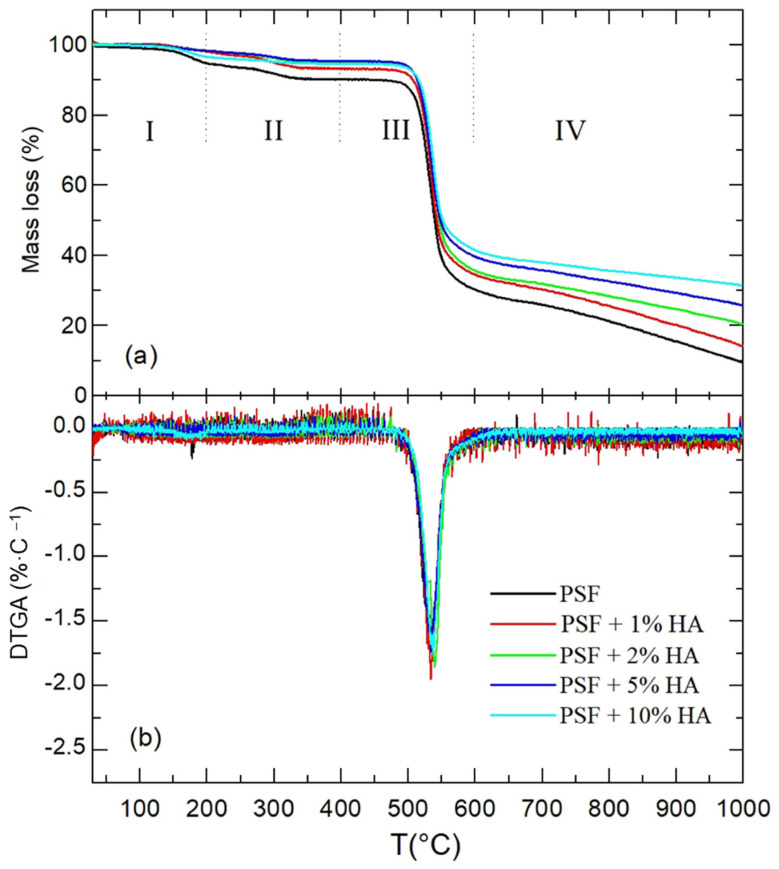
Thermogravimetric analysis, TGA (**a**) and differential thermogravimetric analysis, DTGA (**b**) curves for the PSF and PSF/HA nanocomposites.

The thermal stability of the PSF-based materials was evaluated by thermogravimetric analysis (TGA). Figure 9 shows the TGA and the corresponding differential thermogravimetric analysis, DTGA, curves, for the different materials under study. In general, the pyrolysis of PSF in nitrogen can be divided into different stages. According to Mukhtar and coworkers [40], the first mass loss observed is attributed to the removal of moisture. On the other hand, the next stage observed from 400 °C to 600 °C corresponds to the bulk of the polymer chains degradation, in which the pyrolysis of PSF takes place, being the maximum degradation rate than the one at the peak temperature (T_p_, °C), easily identified in the DTGA curve. After that, there is a final stage that is usually assigned to the slow decomposition of the PSF residue. This residual mass is common in the nitrogen atmosphere, but it can be reduced if the experiment is conducted under air [41,42].

Some parameters determined from TGA and DTGA curves are collected in Table 3. Considering the residual masses, it can be concluded that full thermal decomposition of pure polysulfone is not achieved. Besides, when the concentration of HA nanoparticles increases, the residues increase too but that does not happen as expected in a proportional way. The residual mass is higher than the sum of the expected PSF residue plus the one coming from the HA. Therefore, the presence of the nanoparticles must exert a kind of protective action on the thermal decomposition of the polymer. The slopes of the TGA curves at the final part of the curves (Table 3) decrease when the concentration of HA nanoparticles increases, pointing out a decrease in the thermal degradation rate with the content of the particles. Finally, taking the data of all materials, the average value of T_p_ is 537 ± 3 °C which allows stating that there is not any correlation between the temperature at which faster thermodegradation occurs and the relative amount of nanoparticles.

### 3.4. Mechanical Characterization

**Figure 10:**
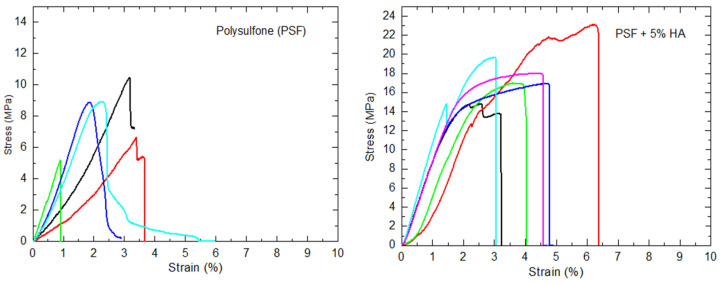
Stress (MPa) vs. strain (%) plots for the samples under study PSF; PSF + 1% HA; PSF + 2%HA; PSF + 5% HA; and PSF +10% HA.

To evaluate the effect of the incorporation of HA particles on the mechanical behavior of the nanocomposites, six specimens of each material were studied by tensile tests. As examples in Figure 10, stress–strain curves obtained for the PSF (left) and PSF filled with 5% HA (right) are presented. The stress–strain plots obtained for the rest of the materials (PSF + 1% HA, PSF + 2% HA, and PSF +10% HA) can be found in the supplementary information (Figures S4–S6, respectively). It can be observed that when PSF is not or little filled (1% and 2%) with HA there is almost no plastic deformation. However, when high enough HA nanoparticles are added (5%, Figure 10, right, and 10%, Figure S6), a clear plastic deformation region is observed in the tensile plots with an increase in the tensile strength which would be translated into an important toughness increase.

Ash et al. [43,44] found an enhancement of ductility in thermoplastic PMMA polymer by adding rigid nanoparticles of alumina. They studied the effect using coated (high compatible coating 3-glycidcoxypropyltrimethoxysilane) and uncoated nanoparticles observing more ductility when uncoated nanoparticles were used and for which agglomeration was identified [44]. They believed the observations were the result of the unique filler characteristics possessed by the nanoparticles leading to a brittle-to-ductile transition at a certain concentration of nanoparticles. Among other reasons, the brittle-to-ductile transitions can be seen by the introduction of residual stresses where the transition is due to the ability of the polymer chains to change their local conformation and relieve the applied triaxial stress before void formation and subsequent crazing can occur [45]. Using this argument, Ash et al. stated that well-dispersed nanoparticles would ensure that the regions of stress-state transformation occur throughout the composite, being the key to observe toughening in many nanocomposite systems. However, when alumina nanoparticles are coated with the most compatible coating, better dispersion of them is expected but the enhancement of ductility was lower. Therefore, other causes must explain the strain to failure increase when a certain number of nanoparticles are added to a brittle thermoplastic polymer like PMMA or, as in the present work, PSF. Here, it is important to highlight that Ash et al. also stated in reference [44] that the brittle-to-ductile transition is found to depend on poor interfacial adhesion between polymer and nanoparticle which would be more in accordance with poorer dispersion of nanoparticles. Considering all the above mentioned, a possible new explanation of the ductility enhancement may be just the formation of nanoparticles agglomerates at a particular concentration and their disruption when the yield point is reached. During the material strain, the polymer chains would be compelled to pass between the particles, favoring polymer confinement, changes of polymer chains, local conformation, and thus stronger intermolecular interactions. This situation would require higher deformations to separate macromolecules from each other to lead to the final mechanical failure of the material.

**Figure 11:**
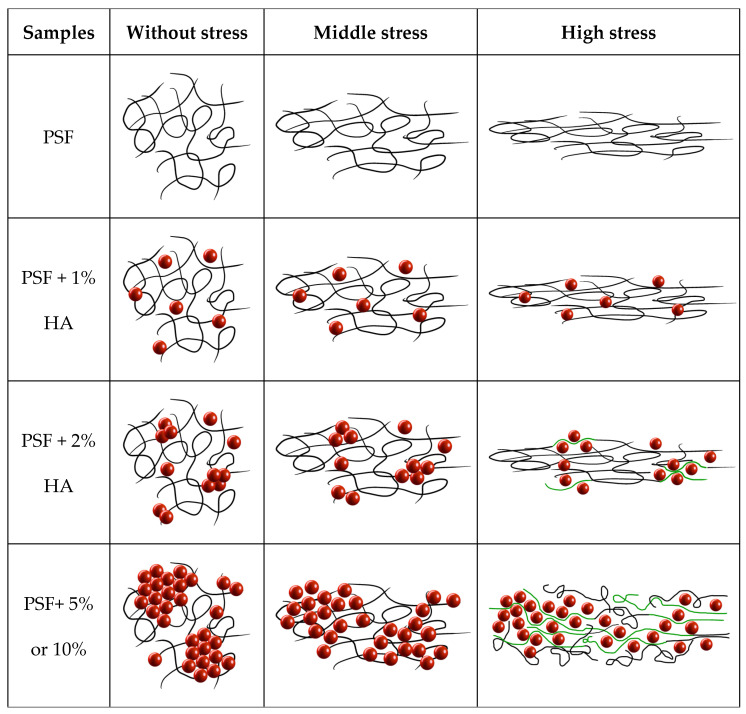
Model explaining the molecular scale behavior of the studied materials under application of uniaxial loads in a tensile test (green lines account for macromolecular portions which conformations are forced to change when nanoparticles rolled away during disaggregation of agglomerates).

The data corresponding to several mechanical parameters extracted from the tensile tests are gathered in Table 4 (tensile strength, elastic modulus, strain to failure, and total area under the tensile curve). In general, tensile strength increases with the concentration of HA nanoparticles. There is only a small decrease in strength (σ) for the sample filled with 2% HA nanoparticles. This behavior observed can be explained considering a balance between three contributions: (a) concentration of nanoparticles; (b) transmission of loads from the polymer matrix to the more mechanically resistant HA nanoparticles; and (c) chains conformational changes and consequently macromolecular arrangement. When uniform dispersion of nanoparticles exists, more efficient transmission of loads is expected and, consequently, the higher the concertation of nanoparticles the higher the tensile strength. However, at a certain concentration of nanoparticles aggregates can be formed reducing the efficiency of load transfer and therefore causing loss of tensile strength. On the other hand, if aggregates or even agglomerates are formed, they can be disaggregated during the tensile test. This aggregates disruption may induce the macromolecules to pass in between the nanoparticles and therefore to force conformation changes that would lead to more ordered arrangements and, consequently, higher interactions between them. These higher intermolecular interactions in materials always correspond to higher mechanical strength. Following the morphology results obtained from STEM observations (Figure 4, Figure 5 and Figure 6), a model can be proposed to explain the mechanism of the strain process in the materials under study (Figure 11).

On the other hand, in general, Young’s modulus increases with particle content which is in accordance with the incorporation of more rigid particles within the system. Again, this slight non-expected decrease in the modulus when the concentration of 2% is considered can be due to a non-efficient transmission of loads when small aggregates of nanoparticles are formed. The data of tensile strength and elastic modulus of PSF prepared by airbrushing in the present work are quite close to those obtained in other works for PSF-based membranes [46].

Finally, the strain to failure follows the same tendency as that of the tensile strength and modulus so as the total area under the curve. All these results can also be explained considering the same three contributions taken into account when tensile strength was explained and making use of the model presented in Figure 11. At a certain concentration of nanoparticles, agglomerates can be disaggregated forcing the nanoparticles to roll away and, consequently, making a pass in between the PSF polymer chains with conformational variations which would allow higher interactions with each other and therefore larger strain before failure.

As a summary, the addition of HA nanoparticles up to 1% leads to an increase in the rigidity of the material in terms of the elastic modulus. These results are in good agreement with the observations given by FTIR. Considering the interpretation extracted from the absorbance band ratios, before any strain of the material the optimum level of PSF/HA interactions is reached when 1% of nanoparticles is added.

When the particle content is 2%, most of the mechanical parameters decrease, which is interpreted in terms of particle aggregation, leading to poor load transfer between the PSF and HA particles. Finally, the improvement in the mechanical properties observed for higher loadings can be associated with an increase in the amount of harder and more rigid material, HA, and the possibility of disaggregation of agglomerates, in which particles may force changes in the PSF chains conformations to subsequently favor intermolecular interactions.

To analyze the effect of HA particles on the elastic modulus of the materials, an estimation of the upper bound (E_upper_) and the lower bound (E_low_) was also done using the rule of mixtures taking into account the density of the pure components (ρ_PSF_ = 1.24 g·cm^−3^; ρ_HA_ = 3.16 g·cm^−3^ [47]). The estimated values of the elastic modulus (E_upper_ and E_low_) were calculated taking the elastic modulus of PSF obtained in the present work (E = 520 MPa) and the elastic modulus of HA taken from the bibliography (E = 52 GPa [48]). The elastic modulus obtained experimentally for the PSF filled with 1% of HA (E_exp_ = 785 MPa) was slightly higher than the estimated E_upp_ (E_upp_ = 723 MPa) which may be a consequence of the efficient reinforcement of the uniformly dispersed particles. However, when the concentration of HA particles increases up to 2%, the experimental modulus was below the estimated one using the rule of mixtures. Again, the consideration of particle aggregation seems to be the most reasonable explanation.

If higher concentrations are considered 5% and 10%, although the aggregates can be as dense as to be considered agglomerates, greater reinforcement was achieved. In terms of Young’s modulus, the result can be simply justified by the simple incorporation of HA harder and more rigid material. However, in terms of tensile strength, the reinforcing effect can only be justified from the effectiveness of load transfer of the particles to the matrix, but in the present work, that event can only happen after the disaggregation of agglomerates as explained before.

On the other hand, in particle-reinforced composite materials, the elastic modulus of the composite, E_c_, can also be estimated using a modified expression of the rule of mixtures, E_c_ = E_m_·V_m_ + K·E_p_·V_p_, where E_m_, E_p_, are the elastic modulus of the matrix and the particles, V_m_, V_p_, the volume fractions of the matrix and particles, and K is a factor that measures the reinforcement efficiency. Considering the experimental results of the elastic modulus obtained in our materials (E_exp_), K values were estimated for each material obtaining the data shown in Table 4. In principle, for the same system using the same type of particles, K should be constant. However, we observe that, as the concentration of HA particles increases, K decreases, i.e., the reinforcing efficiency decreases. Again, here it can be said that this reinforcing parameter drops with the concentration of particles because of aggregates or even agglomerates formation being expected to have the highest efficiency when uniform dispersions of nanoparticles are achieved. K reaches its highest value for the material with 1% of HA nanoparticles for which, before deformation, the highest interactions of polymer–nanoparticles are expected as the FTIR results point out.

Apart from considering the formation of particle aggregates in polymer nanocomposites, the effect of the interphase can also play an important role and can be used to interpret the results. In an attempt to understand the effect of interphases in polymer nanocomposites, we propose the use of a former model developed by J. Gonzalez-Benito et al. in a previous research work [49]. Assuming a close-packing structure of spheres (like an FCC packing) which radii were given by the total radius, R_t_, of both the particle, r, and the interphase, R_i_, the maximum thickness of the interphases that can be achieved was estimated. According to supplier information, the radius of the HA nanoparticles was set to r = 59 nm, and the maximum thickness reachable of the interphase, R_i_ (nm), was left as a fitting parameter which value was estimated as a function of the particle concentration (Table 4). The estimated values ranged between 90–300 nm. According to data published in the literature, the thickness of interphases in composite materials may vary depending on the composition, the interaction between components, and the method used to characterize interphases. For example, J. Gonzalez-Benito et al., [49] using a coefficient of thermal expansion measurements, provided an estimated value of 90 nm for the interphases in EVA/TiO_2_ nanocomposites. E. Mader reported the presence of interphases of 100–200 nm in epoxy and polypropylene glass fiber reinforced composites using AFM nanoindentation measurements [50]. P.K. Agnihotri et al. [51] reported interphases in carbon fiber/epoxy composites ranging from 0.25 µm to 7 µm, measured by scanning electron microscopy (SEM) and EDS analysis. Therefore, all the data in Table 5 are close to those reported in the literature as interphase thicknesses in nanocomposites.

**Figure 12:**
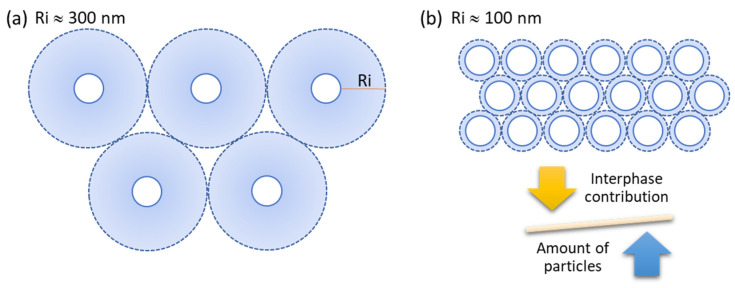
Schematic representation of particles and interphases for two different thicknesses (Ri): (**a**) Ri ≈ 300 nm and (**b**) Ri ≈ 100 nm values like those estimated for PSF + 1% HA and PSF+ 10% HA, respectively.

As expected, when the particle content increases, the maximum possible thickness of the interphase decreases to satisfy the geometrical constraints. To illustrate this, a scheme is shown in Figure 12. However, for the same system, regardless of the concentration of nanoparticles, only one interphase thickness is possible. Therefore, attending the results arising from the STEM and the mechanical tests, the interphase thickness in the materials under study should be higher than 208 nm or the maximum thickness obtained for the sample with 2% of HA nanoparticles (Table 5) or concentration below which uniform dispersion of nanoparticles is achieved. It has been seen that the most effective combination of elastic properties (elastic modulus) is obtained for 1% HA, after that, the modulus drops 2% and then increases 5% and 10% HA. In terms of composite material, it is possible to assume that the interphase itself behaves like a third component with its physicochemical and mechanical properties, and the higher the fraction of interphase, the higher its contribution to enhancing the mechanical properties. Therefore, when a critical concentration of nanoparticles is reached, interphases would overlap, decreasing the global interphase contribution to the mechanical parameter of, in this instance, the Young’s modulus.

In principle, from 2% of HA nanoparticles, an increase in particle content should lead to a modulus increase. Therefore, a balance between two factors (the relative amount of particles and interphase, respectively) should be considered where the decrease in the contribution of the interphase should be more important. This explanation is in good agreement with the decrease in the ‘K–factor’ as particle content increases. Therefore, the ‘K–factor’ is probably related in some way to the interphase contribution in composite materials. Apart from that, when a thermoplastic is used as the matrix in nanocomposites, another contribution should be taken into account in order to interpret the whole mechanical performance; disaggregation of agglomerates that might force macromolecules sliding between nanoparticles with conformational changes favoring intermolecular interactions, which would induce enhancement of strain to failure and mechanical strength.

## 4. Conclusions

In this work, thin films of polysulfone/hydroxyapatite, PSF/HA, nanocomposites were prepared with a commercial airbrush. Mechanical and thermal behavior was interpreted from the information given by structural characterization. In particular, based on the use of a simple model describing the system at a molecular level, it is stated that mechanical properties of PSF/HA nanocomposites are highly conditioned by four highly interrelated factors: nanoparticle dispersion, nanoparticle–polymer interactions, and interphase and nanoparticle concentration.

When HA nanoparticles concentration is low enough (1% wt), uniform dispersion of nanoparticles seems to be achieved, allowing specific interactions of polymer–nanoparticles and appearance of the high fraction of interphase which may contribute to enhance mechanical properties. At a certain concentration (about 2%) nanoparticles aggregates can be formed, decreasing the specific interactions and therefore efficiency of transmission of loads from the nanoparticles to the PSF matrix, besides, interphase overlapping may occur, decreasing its contribution to the mechanical properties. Finally, when the concentration of HA nanoparticles is high enough (5% and 10%) agglomerates of nanoparticles may be formed and when uniaxial stress is applied, they can be disaggregated by rolling away from the nanoparticles which can force the polymer chains to pass in between the nanoparticles causing conformational changes with alignments that would favor macromolecular interactions and consequently result in an increase in strain to failure and tensile strength.

## Figures and Tables

**Table 1:** Band assignment of FTIR spectra for the PSF/HA samples.

Wavelength (cm^−1^)	Band Assignments
3500	OH stretching vibrations
1490–1585	C=C Aromatic stretching vibrations
1322, 1292	S=O asymmetric and symmetric stretching vibrations
1244	C-O-C asymmetric stretching vibration
1151	–C–SO_2_–C– symmetric stretching O=S=O stretching vibration
1103, 1020	Aromatic C–H in-plane bending
1071, 1055	PO_4_^3−^ asymmetric vibrations

**Table 2:** Transition temperatures, T_g_, and changes in the heat capacity, C_p_, determined from the DSC thermograms of the samples under study for the first and second heating scan.

			1st Heating Scan		2nd Heating Scan		
Sample	Mass (mg)	NPs (%,wt)	T_g,1_ (°C)	ΔC_p_ (mW·g_PSF_^−1^)	T_g,2_ (°C)	ΔC_p_ (mW·g_PSF_^−1^)	ΔH_relax_ (J g_PSF_^−1^)
PSF	3.964	0	175.7	54	186.1	57	0.28
PSF + 1%HA	4.110	1	180.7	18	185.6	51	0.32
PSF + 2%HA	4.071	2	180.4	16	185.9	49	0.28
PSF + 5%HA	3.910	5	180.4	22	186.6	51	0.31
PSF + 10%HA	3.937	10	180.1	15	186.4	50	0.27

**Table 3:** Parameters obtained from TGA and DTGA curves (initial mass, residual mass, slope after pyrolysis, and peak temperature, T_p_).

		Initial Mass		Final Mass			DTGA
Sample	Mass (mg)	Mass (mg)	Mass (%)	Mass (mg)	Mass (%)	Slope after Pyrolysis (%·°C^−1^)	T_peak_ (°C)
PSF	0	0.6145	100	0.0596	9.70	−0.04184	540
PSF + 1%HA	0.0567	0.8145	100	0.1163	14.28	−0.04336	532
PSF + 2%HA	0.1128	0.8385	100	0.1724	20.56	−0.03234	541
PSF + 5%HA	0.1989	0.9956	100	0.2585	25.96	−0.03093	534
PSF + 10%HA	0.4937	1.7565	100	0.5533	31.50	−0.02297	536

**Table 4:** Mechanical parameters obtained for the samples of PSF/HA from the mechanical tests.

Sample	σ (MPa)	E_exp_ (MPa)	E_upper_ (MPa)	E_low_ (MPa)	Strain to Failure (%)	Stress to Failure (MPa)	Area (10^6^ J/m^3^)	K
PSF	8.9 ± 3	520 ± 206	520	520	1.03 ± 0.56	8.0 ± 3.4	0.11 ± 0.05	0
PSF + 1%HA	8.6 ± 2	785 ± 179	723	522	0.74 ± 0.26	8.0 ± 2.4	0.08 ±0.05	1.3
PSF + 2%HA	5.8 ± 2	695 ± 182	929	524	0.52 ± 0.12	5.6 ± 1.2	0.03 ± 0.02	0.43
PSF + 5%HA	19.1 ± 3	936 ± 106	1562	531	4.47 ± 1.13	17.9± 2.4	0.55 ± 0.2	0.41
PSF + 10%HA	21.3 ± 4	1160 ± 269	2671	542	5.41 ± 3.79	19.5 ± 6.3	0.76 ± 0.7	0.30

**Table 5:** The estimated thickness of the interphases, Ri, in nanometers, as calculated using the model reported in reference [49].

NPs (%,wt)	Estimated Thickness of the Interphase, Ri (nm)
0	0
1	275
2	208
5	137
10	95

## Data Availability

The data of this study are available upon request.

## Supplementary Materials

The following supporting information can be downloaded at: https://www.mdpi.com/article/10.3390/polym14040753/s1, Figure S1 X-ray diffraction pattern of the commercial hydroxyapatite powder (according to standard of JCPDS: 9-432) [52,53]; Figure S2 Particle size distributions obtained for hydroxyapatite nanoparticles with spherical morphology (a) and with rod-like morphology for the length (b) and width (c) of the nanoparticles; Figure S3 (Left) Zoom region of FTIR spectra and (right) band ratio for PSF/HA nanocomposites; Figure S4 Stress (MPa) vs. strain (%) curve of PSF + 1% HA nanocomposites; Figure S5 Stress (MPa) vs. strain (%) curve of PSF + 2% HA nanocomposites; and Figure S6 Stress (MPa) vs. strain (%) curve of PSF + 10% HA nanocomposites.
