# Supplementary material for: Airbrushed Polysulfone (PSF)/Hydroxyapatite (HA) Nanocomposites: Effect of the Presence of Nanoparticles on Mechanical Behavior
Source: Polymers (Basel). 2022 Feb 15;14(4):753. doi: 10.3390/polym14040753 (PMC8878103; doi:10.3390/polym14040753)
Supplement: Supplementary file 1 [file polymers-14-00753-s001.zip › polymers-1538768-supplementary.pdf]

polymers

MDPI

Article

Airbrushed Polysulfone (PSF)/Hydroxyapatite (HA) Nanocomposites: Effect of The Presence of Nanoparticles on The Mechanical Behaviour

Monireh Moradienayat, Dania Olmos * and Javier González-Benito *

Universidad Carlos III de Madrid, Departamento de Ciencia e Ingeniería de Materiales e Ingeniería Química, IQMAAB, Avda. de la Universidad 30, 28911 Leganés, Spain 1; mmoradie@ing.uc3m.es

* Correspondence: D.O.: dolmos@ing.uc3m.es; J.G-B: javid@ing.uc3m.es Tel.: (optional; 28911; if there are multiple corresponding authors, add author initials)

2. Materials and Methods

2.1. Materials

In Figure S1, XRD of the HA nanoparticles is presented. The crystalline phase structure of hydroxyapatite is depicted in the XRD spectrum shown in **Figure S1**. The sharp XRD peaks at 27.3°, 28.63°, 31.96°, 33.23°, 34.30°, 40.22°, 44.23°, 46.70°, and 52.72° indicate the crystalline phases of HA [1,2].

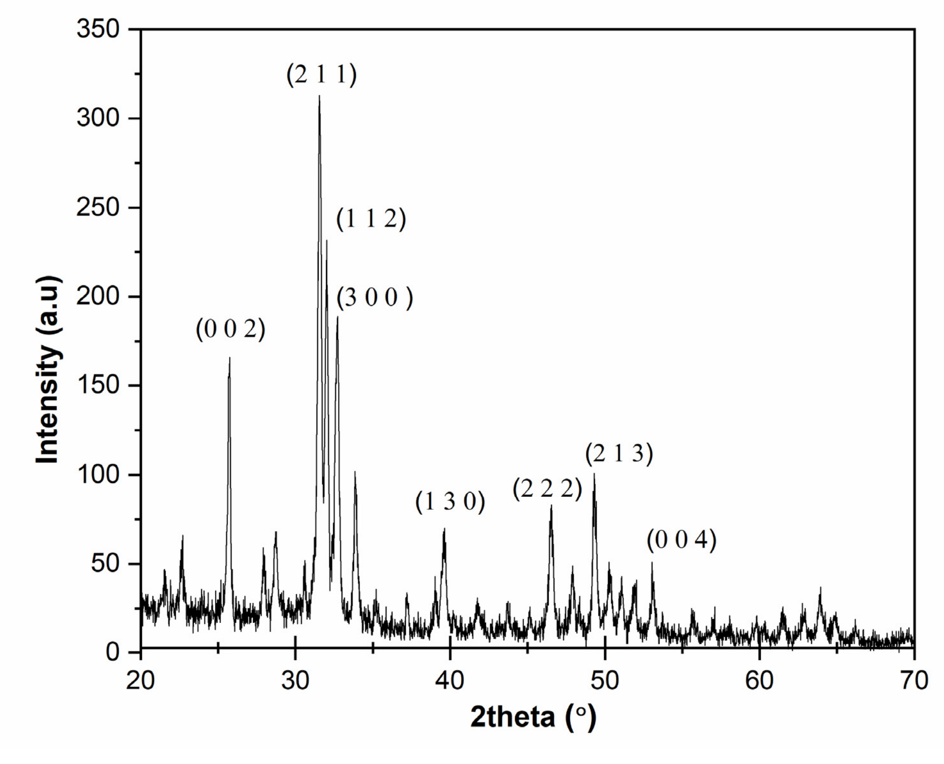

Figure S1. X-ray diffraction pattern of the commercial hydroxyapatite powder (according to standard of JCPDS: 9-432)

In Figure S2, particle size distributions obtained from SEM micrographs for hydroxyapatite nanoparticles. Figure S2a shows particle size distribution for the nanoparticles with spherical morphology which average particle size is 68 nm. For rod-like nanoparticles, similar size distributions were calculated to evaluate the average length (Figure S2b)

and width (Figure S2c), obtaining an average length of 102 ± 27 nm and an average width of 36 ± 11 nm. According to BET characterization, the average particle size of the nanoparticles would be approximately 118 nm (personal communication from the supplier), consistent with our results and with the information given by the supplier (particle size of HA less than 200 nm).

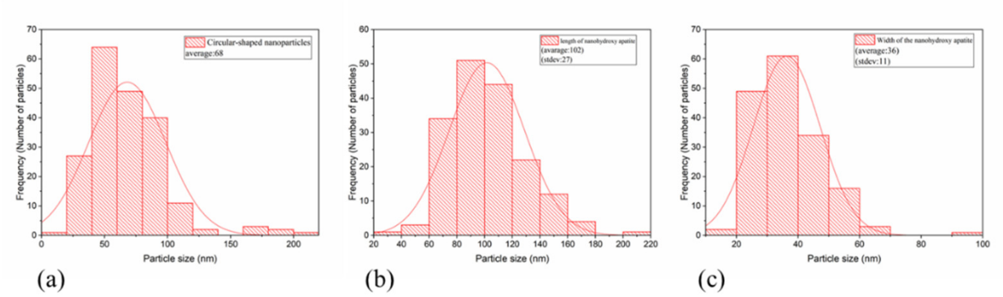

Figure S2. Particle size distributions obtained for hydroxyapatite nanoparticles with spherical morphology (a) and with rod-like morphology for the length (b) and width (c) of the nanoparticles

3. Results

3.1. Structural characterization

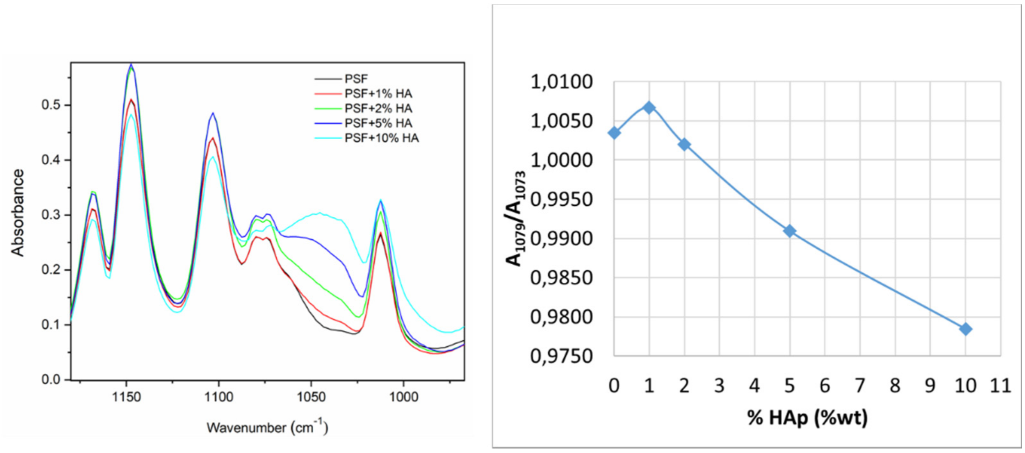

Figure S3. (Left) Zoom region of FTIR spectra and (right) band ratio for PSF/HA nanocomposites

3.4. Mechanical characterization

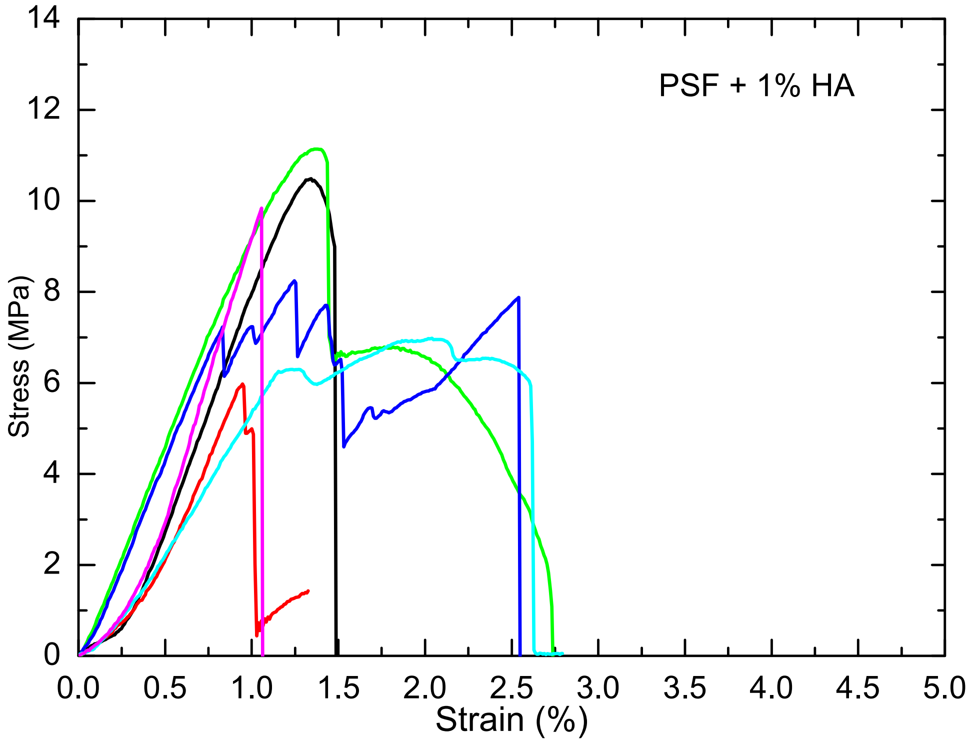

Figure S4. Stress (MPa) vs strain (%) curve of PSF + 1% HA nanocomposites.

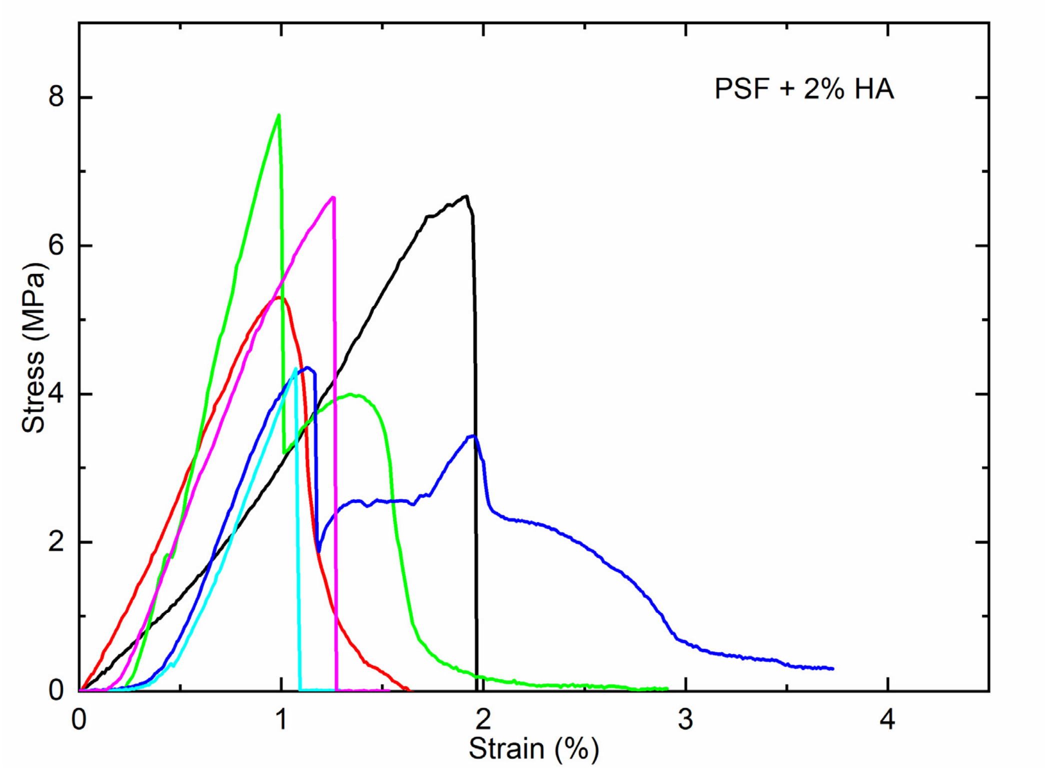

Figure S5. Stress (MPa) vs strain (%) curve of PSF + 2% HA nanocomposites.

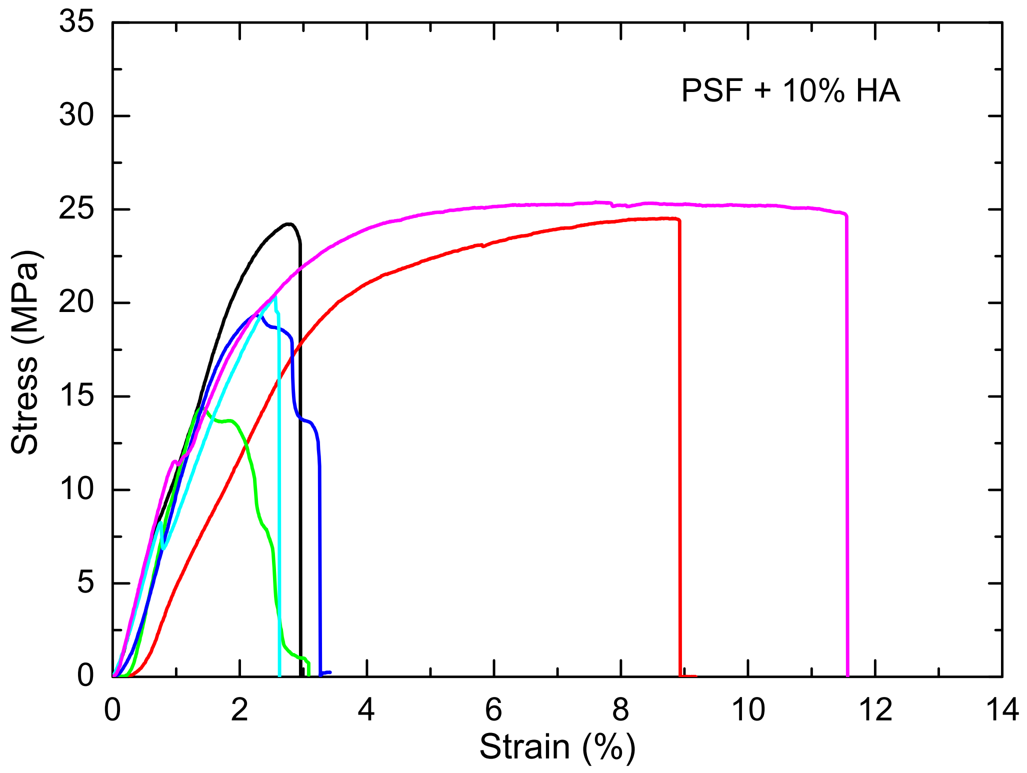

Figure S6. Stress (MPa) vs strain (%) curve of PSF + 10% HA nanocomposites.

References

1. Brundavanam, R.K.; Eddy, G.; Poinern, J.; Fawcett, D. Modelling the Crystal Structure of a 30 nm Sized Particle based Hydroxyapatite Powder Synthesised under the Influence of Ultrasound Irradiation from X-ray powder Diffraction Data. *Am. J. M Aterials Sci.* **2013**, *3*, 84–90. https://doi.org/10.5923/j.materials.20130304.04.
2. Chandrasekar, A.; Sagadevan, S.; Dakshnamoorthy, A. Synthesis and characterization of nano-hydroxyapatite (n-HAP) using the wet chemical technique. *Int. J. Phys. Sci.* **2013**, *8*, 1639–1645. https://doi.org/10.5897/IJPS2013.3990.
